# Legal innovations for balancing environmental protection and public health in urban polluted areas

**DOI:** 10.3389/fpubh.2025.1557173

**Published:** 2025-05-30

**Authors:** Jinglin Zhao, Ruolin Zhang

**Affiliations:** Law School, Xi'an Jiaotong University, Xi'an, Shaanxi, China

**Keywords:** urban pollution, environmental protection, public health, legal frameworks, adaptive interventions, resilience, governance, equity

## Abstract

**Introduction:**

Balancing environmental protection with public health in urban polluted areas presents significant governance and legal challenges. Traditional regulatory approaches often operate in silos, failing to integrate environmental sustainability with health policies, leading to inefficiencies and inequitable outcomes.

**Methods:**

This study introduces the Resilient Ecosystem Management Framework (REMF), an interdisciplinary approach that combines legal innovations, adaptive governance mechanisms, and data-driven environmental health strategies. It critically analyzes the limitations of existing legal frameworks in addressing urban pollution and associated health risks. REMF is developed by integrating adaptive legal instruments, participatory governance, and real-time environmental monitoring. Case studies and quantitative modeling are applied to evaluate the framework's effectiveness in urban environments.

**Results:**

The findings demonstrate that REMF enhances policy coordination, enables more effective regulatory enforcement, and improves environmental and health outcomes by leveraging legal adaptability and technological advancements. Real-time environmental data and predictive analytics allow for dynamic adjustments to legal thresholds, ensuring responsive and equitable governance.

**Discussion:**

Moreover, the framework facilitates active stakeholder engagement, ensuring that vulnerable populations benefit from pollution mitigation efforts. This research provides a scalable and replicable legal model that aligns environmental law with public health imperatives, offering practical insights for policymakers, urban planners, and environmental regulators. By demonstrating how legal frameworks can evolve to address contemporary urban challenges, this study contributes to the broader discourse on sustainable urban governance and environmental justice.

## 1 Introduction

Urban areas worldwide face the dual challenge of environmental degradation and public health crises, primarily driven by air pollution, water contamination, and industrial waste (1). Balancing environmental protection and public health requires innovative legal frameworks that address both issues without compromising economic development and urban growth (2). Traditional legal mechanisms often focus on either environmental protection or public health, leading to fragmented policies that fail to address the intersection of the two domains comprehensively (3). This disconnect not only limits the efficacy of such frameworks but also exacerbates the vulnerability of urban populations to pollution-related diseases and environmental degradation (4). Legal innovations in this area aim to bridge this gap by creating integrated frameworks that align environmental sustainability with public health priorities (5). Such frameworks can promote long-term urban resilience by ensuring cleaner environments while simultaneously safeguarding human health through enforceable regulations and adaptive governance mechanisms.

Early legal approaches to environmental protection in urban polluted areas relied on command-and-control regulations (6). These included prescriptive standards for pollutant emissions, mandatory environmental impact assessments (EIAs), and the establishment of protected zones to limit industrial encroachment on residential areas (7). Although these laws laid the foundation for addressing environmental concerns, they often failed to account for their impact on public health (8). emission caps for industries were based on ecological thresholds rather than health-related outcomes, leading to persistent respiratory and cardiovascular health issues among urban populations (9). these rigid frameworks lacked flexibility, making it challenging to adapt to evolving environmental and public health challenges (10). The inefficiency of these traditional methods highlighted the need for more dynamic and interdisciplinary legal approaches that integrate environmental and health considerations.

In response to the limitations of command-and-control approaches, market-based mechanisms and participatory legal frameworks emerged as a second wave of innovations (11). These included tools such as pollution trading schemes, green taxes, and public-private partnerships (PPPs) aimed at incentivizing businesses and urban stakeholders to adopt sustainable practices (12). Cap-and-trade systems allowed polluting entities to buy and sell emission allowances, promoting cost-effective pollution reduction (13). participatory mechanisms, such as citizen-led lawsuits and community involvement in urban planning, increased public accountability and transparency (14). While these methods introduced economic incentives and democratized environmental decision-making, they often struggled to deliver equitable outcomes (15). Vulnerable populations in highly polluted urban areas frequently lacked the resources to participate in these mechanisms, resulting in an unequal distribution of environmental and health benefits. market-based tools sometimes prioritized economic efficiency over health outcomes, further underscoring the need for holistic legal solutions.

The most recent wave of legal innovations has focused on integrated frameworks that explicitly link environmental protection with public health (16). These frameworks leverage advancements in technology, data analytics, and collaborative governance to create adaptive legal mechanisms (17). smart cities have introduced real-time air quality monitoring systems linked to legal thresholds for pollution control, enabling governments to impose immediate penalties on violators (18). health impact assessments (HIAs) are increasingly being integrated into environmental policymaking, ensuring that urban development projects are evaluated not only for their ecological impact but also for their public health implications (19). Another example is the use of legal tools to promote nature-based solutions, such as urban green spaces and wetland restoration, which simultaneously mitigate pollution and enhance public health by reducing heat island effects and improving mental well-being. these innovations face challenges related to enforcement, data privacy, and the unequal distribution of technological infrastructure in urban areas, which can perpetuate existing disparities (20).

Given the limitations of existing legal frameworks, we propose a novel legal model for balancing environmental protection and public health in urban polluted areas. This model integrates adaptive legal instruments, participatory governance, and real-time data-driven approaches to create a comprehensive solution. By incorporating health-focused environmental standards, community engagement, and technology-enabled monitoring systems, this framework ensures equitable and effective outcomes that address both environmental and public health concerns.

- Introduces a legal framework that combines environmental and public health standards with adaptive legal instruments and participatory governance.
- Ensures vulnerable populations in urban areas benefit from the framework through community involvement and equitable distribution of resources.
- Utilizes real-time monitoring and data analytics to enforce regulations and adapt policies to dynamic urban challenges effectively.

## 2 Related work

### 2.1 Legal frameworks for environmental health governance

Legal frameworks play a critical role in managing the intersection of environmental protection and public health, particularly in urban areas characterized by high levels of pollution (21). Effective governance in this domain requires laws and regulations that address the dual challenges of safeguarding environmental resources and protecting vulnerable populations from health risks. Over the years, various countries have adopted innovative legal mechanisms that integrate environmental and health considerations into urban policy-making (22). These frameworks often encompass pollution control standards, land-use planning, and public health monitoring systems. Environmental health governance is increasingly guided by principles such as the precautionary principle and the polluter-pays principle (23). The precautionary principle emphasizes preventive measures to avoid environmental degradation and health hazards, even in the absence of scientific certainty (24). This principle has been incorporated into international agreements like the Rio Declaration on Environment and Development and domestic legislation in several jurisdictions. the polluter-pays principle assigns financial responsibility for pollution control to the entities responsible for environmental harm, incentivizing compliance with regulatory standards. Urban areas with high industrial activity, such as those in developing countries, have particularly benefited from these principles when integrated into local governance structures. Emerging trends in this field include the use of environmental health impact assessments (EHIAs) to evaluate the implications of proposed urban development projects (25). Unlike traditional environmental impact assessments, EHIAs explicitly consider public health outcomes, providing a more comprehensive understanding of how pollution affects human well-being. legal mandates requiring cross-sectoral collaboration between environmental agencies and public health departments have proven effective in addressing complex challenges in urban settings (26). By aligning goals and resources, these collaborative approaches ensure that environmental protection measures also contribute to improved health outcomes, creating a balanced strategy for sustainable urban development (26).

### 2.2 Innovative legal instruments for pollution mitigation

Addressing pollution in urban areas requires legal instruments that go beyond traditional command-and-control regulations (27). Innovative mechanisms such as market-based instruments, public-private partnerships (PPPs), and technology mandates have emerged as effective tools for mitigating pollution while promoting public health (28). Market-based instruments, including carbon pricing, emissions trading systems, and pollution taxes, provide economic incentives for reducing environmental harm. These tools encourage industries to adopt cleaner technologies and minimize emissions, ultimately benefiting urban populations exposed to air and water pollution. Public-private partnerships have also gained traction as a means of addressing urban pollution (29). These partnerships leverage the resources and expertise of private entities to complement government efforts in environmental protection (30). PPPs have been successfully employed to develop green infrastructure projects, such as urban forests and wastewater treatment facilities, which mitigate pollution and enhance public health. Legal frameworks that facilitate PPPs often include provisions for transparent contract negotiation, risk-sharing, and performance monitoring to ensure accountability and effectiveness. legal mandates for adopting clean technologies have driven significant advancements in pollution control (31). Examples include the enforcement of vehicle emissions standards, the promotion of renewable energy sources, and the phasing out of hazardous materials in industrial processes. These mandates not only reduce pollution levels but also create co-benefits for public health by decreasing exposure to harmful substances. As urban areas face increasing pressure to address climate change and environmental degradation, the integration of such innovative legal instruments into urban policies will be essential for balancing environmental protection and health outcomes.

### 2.3 Community-centric legal approaches to urban health

Community engagement is a cornerstone of effective legal strategies for balancing environmental protection and public health in urban areas (32). Recognizing the disproportionate impact of pollution on marginalized communities, recent legal innovations have emphasized the importance of participatory governance and access to justice (33). Laws and policies that empower communities to participate in environmental decision-making not only enhance transparency and accountability but also ensure that public health priorities are addressed in a socially equitable manner. One significant legal innovation in this area is the concept of environmental justice, which seeks to address the unequal distribution of environmental risks and benefits (34). Environmental justice frameworks often include provisions for community involvement in the planning and implementation of pollution control measures (35). some jurisdictions have established community advisory boards and public consultation requirements as part of their environmental governance processes. These mechanisms enable residents to voice concerns, influence policy decisions, and monitor compliance with environmental and health standards. Access to justice is another critical component of community-centric legal approaches (36). Legal systems that provide communities with the ability to challenge polluters and government agencies through administrative, civil, or criminal proceedings have proven effective in addressing environmental health disparities (37). citizen suits have been instrumental in enforcing air and water quality standards in urban areas with high levels of industrial pollution. Legal aid services and public interest litigation further enhance access to justice by removing financial and procedural barriers for disadvantaged populations. The integration of traditional knowledge and cultural values into legal frameworks also represents a community-centric approach to urban health. In many cases, indigenous and local communities possess valuable knowledge about sustainable resource management and environmental protection. Legal recognition of these perspectives not only enriches urban governance but also strengthens community resilience to environmental and health challenges. By adopting participatory and inclusive legal strategies, urban areas can better address the complex interplay between environmental protection and public health.

Green credit policies have emerged as a key financial mechanism to promote environmental sustainability while influencing public health outcomes (38). These policies use financial incentives, such as preferential loans and credit constraints, to encourage businesses to adopt environmentally friendly practices and reduce pollution. A growing body of literature has examined the impact of green finance on environmental improvements and its indirect effects on human health. For instance, recent studies, including Green Credit Policy and Residents' Health: Quasi-Natural Experimental Evidence from China, highlight how financial regulations can drive pollution reduction and subsequently improve public health. By imposing stricter credit access conditions on high-polluting industries, green credit policies effectively reduce industrial emissions, leading to improved air and water quality. Empirical evidence suggests that regions implementing stringent green credit regulations experience lower incidences of respiratory diseases and cardiovascular conditions, demonstrating a clear link between financial policy and health outcomes. While green credit policies play a significant role in environmental governance, they have limitations. their effectiveness depends on enforcement and financial institutions' willingness to prioritize sustainability over short-term profitability. these policies primarily target industrial pollution sources, often neglecting urban pollution from transportation, residential waste, and other non-industrial sources that also contribute to public health risks. the unequal distribution of green financing may lead to disparities, where well-funded enterprises can adapt more easily than smaller businesses, potentially exacerbating socio-economic inequalities. In comparison, our study proposes the Resilient Ecosystem Management Framework (REMF), which extends beyond financial tools to integrate legal adaptability, participatory governance, and real-time environmental monitoring. Unlike green credit policies, which rely on indirect incentives, our framework directly aligns legal interventions with public health objectives, ensuring a more responsive and equitable approach. By incorporating real-time data analysis and adaptive legal thresholds, REMF allows for continuous adjustments in pollution control measures, whereas financial policies like green credit often operate on longer policy cycles with delayed impacts. by engaging multiple stakeholders–including government agencies, businesses, and communities–our model ensures that regulatory changes are inclusive and adaptable to diverse urban environments.

## 3 Method

### 3.1 Overview

Environmental protection has become an urgent global priority, demanding innovative strategies and interdisciplinary approaches to mitigate the impact of human activities on ecosystems. This subsection provides a comprehensive overview of the methodologies and objectives driving our work on environmental protection. we focus on integrating data-driven models, advanced simulation frameworks, and adaptive intervention strategies to address critical environmental challenges such as pollution control, resource conservation, and ecosystem restoration.

This paper is structured around three foundational components, detailed in Preliminaries, Sustainable Environmental Impact Model (SEIM), and Resilient Ecosystem Management Framework (REMF). we present the Preliminaries, where we formalize key problems in environmental protection by introducing mathematical frameworks and systemic models to represent environmental dynamics and stressors. This section sets the foundation for understanding the complexity of environmental processes and the need for targeted interventions. we introduce a novel modeling approach, termed the Sustainable Environmental Impact Model (SEIM), which leverages predictive analytics and real-time data to simulate the effects of environmental stressors and evaluate the outcomes of various mitigation strategies. In the Resilient Ecosystem Management Framework (REMF), REMF emphasizes aligning environmental policies with public health goals through dynamic and interdisciplinary measures. REMF leverages real-time environmental data, predictive analytics, and participatory governance to create scalable solutions capable of responding to the evolving challenges of urban ecosystems. It also provides a pathway for promoting health equity by ensuring that vulnerable populations benefit from pollution reduction measures.

### 3.2 Preliminaries

Environmental protection involves the design and implementation of strategies to mitigate the adverse impacts of human activity on ecosystems, conserve natural resources, and ensure the sustainability of environmental systems. This section formalizes the complex dynamics of environmental systems through mathematical frameworks that capture the interactions between anthropogenic activities, natural processes, and environmental outcomes. These formulations serve as the foundation for developing predictive models and intervention strategies to address pressing environmental challenges.

Human activities such as industrial emissions, deforestation, and agricultural practices exert significant pressure on the environment. Let **A**(*t*) denote the vector of anthropogenic activities, with elements *A*_*k*_(*t*) representing the intensity of the *k*-th activity. The impact of these activities on the environment is modeled using a coupling matrix **C**, where *C*_*ij*_ quantifies the influence of activity *A*_*i*_(*t*) on environmental indicator *E*_*j*_(*t*):


(1) $$\begin{array}{l} \text{E}\left(t\right)=\text{C}\cdot \text{A}\left(t\right)+\text{N}\left(t\right), \end{array}$$

where **N**(*t*) captures the contributions of natural processes such as weather patterns and ecosystem resilience.

Pollution dynamics are critical to understanding environmental degradation. Let *P*(*x, y, t*) represent the pollutant concentration at spatial location (*x, y*) and time *t*. The temporal evolution of *P*(*x, y, t*) is governed by the advection-diffusion equation:


(2) $$\begin{array}{l} \frac{\partial P(x,y,t)}{\partial t}+v(x,y,t)\cdot \nabla P(x,y,t)=D\;\nabla^{2}P(x,y,t)+S(x,y,t)\\ \;-\;R(x,y,t), \end{array}$$

where: - **v**(*x, y, t*) is the velocity field (e.g., wind or water currents), - *D* is the diffusion coefficient, - *S*(*x, y, t*) is the source term representing pollutant emissions, - *R*(*x, y, t*) is the removal rate due to natural or artificial processes.

Natural resource dynamics are modeled to evaluate the sustainability of resource extraction and consumption. Let *R*(*t*) denote the availability of a particular resource, which evolves according to the balance between extraction *E*(*t*), natural replenishment *G*(*t*), and degradation *D*(*t*):


(3) $$\begin{array}{l} \frac{dR\left(t\right)}{dt}=G\left(t\right)-E\left(t\right)-D\left(t\right). \end{array}$$

For renewable resources, *G*(*t*) is often a logistic growth function:


(4) $$\begin{array}{l} G\left(t\right)=rR\left(t\right)\left(1-\frac{R\left(t\right)}{K}\right), \end{array}$$

where *r* is the intrinsic growth rate, and *K* is the carrying capacity of the environment.

Ecosystem health and biodiversity are integral to environmental stability. Biodiversity indices *B*_*i*_(*t*) are modeled as functions of habitat quality *Q*_*h*_(*t*), population dynamics *N*_*i*_(*t*), and stress factors *S*_*i*_(*t*):


(5) $$\begin{array}{l} \frac{dB_{i}\left(t\right)}{dt}=f\left(Q_{h}\left(t\right),N_{i}\left(t\right)\right)-S_{i}\left(t\right). \end{array}$$

Habitat quality *Q*_*h*_(*t*) is influenced by land use changes, *L*(*t*), and conservation efforts, *C*_*h*_(*t*):


(6) $$\begin{array}{l} Q_{h}\left(t\right)=Q_{h}^{0}-\alpha L\left(t\right)+\beta C_{h}\left(t\right), \end{array}$$

where $Q_{h}^{0}$ is the initial habitat quality, and α, β are scaling factors.

To mitigate environmental impacts, targeted interventions are introduced. Let *I*(*t*) represent the vector of intervention efforts, such as pollution control technologies, afforestation programs, and renewable energy adoption. The adjusted environmental dynamics are given by:


(7) $$\begin{array}{l} \text{E}\left(t\right)=\text{C}\cdot \text{A}\left(t\right)-\text{M}\cdot \text{I}\left(t\right)+\text{N}\left(t\right), \end{array}$$

where **M** is the mitigation efficiency matrix, with *M*_*ij*_ quantifying the effectiveness of intervention *I*_*i*_(*t*) on indicator *E*_*j*_(*t*).

Environmental protection often involves balancing multiple objectives, such as reducing pollution, conserving resources, and maintaining economic viability. Let $O=\left\{O_{1},O_{2},\ldots ,O_{k}\right\}$ denote the set of objectives. The optimization problem is formulated as:


(8) $$\begin{array}{l} \underset{\text{I}\left(t\right)}{\max}\;F\left(O\right)=\overset{k}{\underset{i=1}{\sum }}w_{i}O_{i}, \end{array}$$

subject to budget constraints $\overset{n}{\underset{i=1}{\sum }}C_{i}I_{i}\left(t\right)\leq B$, where *w*_*i*_ are weights reflecting the priority of each objective, and *B* is the total budget.

Combining the above components, the integrated model for environmental protection is expressed as:


(9) $$\begin{array}{l} \frac{d\text{E}\left(t\right)}{dt}=\text{F}\left(\text{E}\left(t\right),\text{A}\left(t\right),\text{I}\left(t\right),\text{N}\left(t\right)\right), \end{array}$$

where **F** encapsulates the coupled dynamics of environmental indicators, anthropogenic activities, and mitigation strategies.

### 3.3 Sustainable environmental impact model (SEIM)

**Figure 1:**
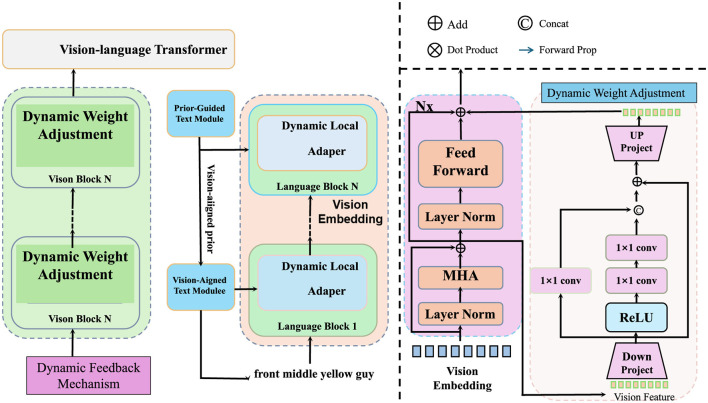
Architecture of the Sustainable Environmental Impact Model (SEIM), illustrating dynamic weight adjustment, layered modeling framework, and vision-language integration to quantify and mitigate environmental impacts.

address the multifaceted challenges of environmental protection, we propose a novel modeling framework, termed the Sustainable Environmental Impact Model (SEIM). SEIM integrates data-driven predictions, dynamic system modeling, and multi-scale environmental interactions to quantify the impacts of human activities and guide mitigation strategies. This section introduces the structure, components, and mathematical formulation of SEIM, emphasizing its capacity to capture complex interdependencies across environmental, anthropogenic, and systemic factors (as shown in Figure 1).

#### 3.3.1 Layered modeling framework with interaction mapping

SEIM is designed to model environmental systems through three interconnected layers. The Anthropogenic Activity Layer represents human activities and their direct environmental impacts. The Environmental Dynamics Layer models the evolution of environmental indicators based on natural processes and external stressors. The Mitigation and Adaptation Layer simulates the effects of intervention strategies to reduce or offset environmental damage.

Let the state of the environment at time *t* be represented by the vector **E**(*t*), as defined in the preliminaries, while **A**(*t*) denotes anthropogenic activities and **I**(*t*) intervention efforts. SEIM uses a system of coupled differential equations to capture the interactions between these layers. The influence of human activities on environmental indicators is represented through a linear mapping:


(10) $$\begin{array}{l} \frac{d\text{E}\left(t\right)}{dt}=\text{C}\cdot \text{A}\left(t\right), \end{array}$$

where **C** is the activity-to-impact matrix, as introduced earlier. The elements of **C**, denoted *C*_*ij*_, quantify the sensitivity of environmental indicator *E*_*j*_(*t*) to activity *A*_*i*_(*t*). industrial emissions *A*_ind_(*t*) contribute to air pollutant concentrations *P*(*t*):


(11) $$\begin{array}{l} \frac{dP\left(t\right)}{dt}=C_{P,\text{ind}}A_{\text{ind}}\left(t\right). \end{array}$$

To account for nonlinear interactions, **C** can be expanded to include higher-order terms:


(12) $$\begin{array}{l} \frac{d\text{E}\left(t\right)}{dt}=\text{C}\cdot \text{A}\left(t\right)+{\text{C}}^{\prime }\cdot \text{A}\left(t\right)⊗\text{A}\left(t\right), \end{array}$$

where ⊗ denotes the outer product, and **C**′ captures cross-dependencies between activities.

Mitigation and adaptation efforts are included through an additive term involving **I**(*t*):


(13) $$\begin{array}{l} \frac{d\text{E}\left(t\right)}{dt}=\text{C}\cdot \text{A}\left(t\right)+{\text{C}}^{\prime }\cdot \text{A}\left(t\right)⊗\text{A}\left(t\right)-\text{M}\cdot \text{I}\left(t\right), \end{array}$$

where **M** is the mitigation matrix, whose elements *M*_*kj*_ represent the effectiveness of intervention *I*_*k*_(*t*) in reducing the stress on environmental indicator *E*_*j*_(*t*).

The evolution of anthropogenic activities is modeled as:


(14) $$\begin{array}{l} \frac{d\text{A}\left(t\right)}{dt}=-\text{K}\cdot \text{A}\left(t\right)+\text{D}\cdot \text{E}\left(t\right), \end{array}$$

where **K** represents decay or regulation factors reducing activities over time, and **D** captures feedback effects from environmental degradation on human activities.

The intervention strategies are modeled through dynamic adaptation, where:


(15) $$\begin{array}{l} \frac{d\text{I}\left(t\right)}{dt}=\text{N}\cdot \text{E}\left(t\right)-\text{L}\cdot \text{I}\left(t\right), \end{array}$$

with **N** representing the responsiveness of interventions to environmental states, and **L** being a decay matrix reflecting the diminishing effect of interventions over time.

The complete coupled system of the SEIM framework integrates these components:


(16) $$\begin{array}{l} \frac{d}{dt}\left[\begin{array}{c} \text{E}\left(t\right)\\ \text{A}\left(t\right)\\ \text{I}\left(t\right) \end{array}\right]=\left[\begin{array}{ccc} \text{C} & \text{0} & -\text{M}\\ \text{D} & -\text{K} & \text{0}\\ \text{N} & \text{0} & -\text{L} \end{array}\right]\cdot \left[\begin{array}{c} \text{E}\left(t\right)\\ \text{A}\left(t\right)\\ \text{I}\left(t\right) \end{array}\right]+\left[\begin{array}{c} {\text{C}}^{\prime }\cdot \text{A}\left(t\right)⊗\text{A}\left(t\right)\\ \text{0}\\ \text{0} \end{array}\right]. \end{array}$$

This final equation captures the comprehensive dynamics of the layered framework, including direct, nonlinear, and feedback interactions between anthropogenic activities, environmental dynamics, and mitigation efforts.

#### 3.3.2 Dynamic environmental system modeling

Environmental indicators evolve under the combined influence of anthropogenic activities, natural processes, and feedback mechanisms. The dynamics are governed by a system of coupled differential equations:


(17) $$\begin{array}{l} \frac{d\text{E}\left(t\right)}{dt}={\text{F}}_{E}\left(\text{E}\left(t\right),\text{A}\left(t\right)\right), \end{array}$$

where **F**_*E*_ encapsulates processes such as pollution dispersion, resource depletion, and biodiversity loss. For a pollutant *P*(*x, y, t*), SEIM incorporates the advection-diffusion equation:


(18) $$\begin{array}{l} \frac{\partial P(x,y,t)}{\partial t}+v(x,y,t)\cdot \nabla P(x,y,t)=D\nabla^{2}P(x,y,t)+S(x,y,t)\\ \;-\;R(x,y,t), \end{array}$$

where *S*(*x, y, t*) is the source term from anthropogenic emissions, and *R*(*x, y, t*) represents the removal rate via natural or artificial processes.

For renewable resources *R*(*t*), the model integrates extraction, replenishment, and degradation:


(19) $$\begin{array}{l} \frac{dR\left(t\right)}{dt}=rR\left(t\right)\left(1-\frac{R\left(t\right)}{K}\right)-E\left(t\right)-D\left(t\right), \end{array}$$

where *r* is the intrinsic growth rate, *K* the carrying capacity, *E*(*t*) the extraction rate, and *D*(*t*) the degradation rate. Biodiversity *B*(*t*) is modeled as a function of habitat quality *Q*_*h*_(*t*), species populations *N*(*t*), and stress factors *S*(*t*):


(20) $$\begin{array}{l} \frac{dB\left(t\right)}{dt}=\gamma Q_{h}\left(t\right)N\left(t\right)-\delta S\left(t\right), \end{array}$$

where γ and δ are scaling factors.

To simulate feedback mechanisms, the model includes an environmental stress function **S**_*E*_(*t*), which reflects the cumulative impact of anthropogenic activities and environmental degradation:


(21) $$\begin{array}{l} {\text{S}}_{E}\left(t\right)=\int_{0}^{t}\alpha \text{A}\left(t^{\prime }\right)+\beta \text{E}\left(t^{\prime }\right)dt^{\prime }, \end{array}$$

where α and β represent the contributions of anthropogenic activities and environmental indicators to stress accumulation.

The interaction between species population *N*_*i*_(*t*) and biodiversity is governed by a Lotka-Volterra-type equation:


(22) $$\begin{array}{l} \frac{dN_{i}\left(t\right)}{dt}=N_{i}\left(t\right)\left(r_{i}-\overset{n}{\underset{j=1}{\sum }}c_{ij}N_{j}\left(t\right)\right)-h_{i}\left(t\right), \end{array}$$

where *r*_*i*_ is the intrinsic growth rate of species *i*, *c*_*ij*_ are interspecies competition coefficients, and *h*_*i*_(*t*) reflects anthropogenic impacts on species *i*.

Carbon dynamics are modeled through the net flux *C*(*t*) in the atmosphere, balancing emissions, absorption, and decay:


(23) $$\begin{array}{l} \frac{dC\left(t\right)}{dt}=E_{C}\left(t\right)-A_{C}\left(t\right)-D_{C}\left(t\right), \end{array}$$

where *E*_*C*_(*t*) is the emission rate, *A*_*C*_(*t*) is the absorption rate by natural sinks, and *D*_*C*_(*t*) is the decay rate due to mitigation efforts.

Water quality *W*(*t*) is affected by pollutant inflows *I*_*P*_(*t*), self-purification processes *P*_*W*_(*t*), and human interventions *M*_*W*_(*t*):


(24) $$\begin{array}{l} \frac{dW\left(t\right)}{dt}=-I_{P}\left(t\right)+P_{W}\left(t\right)-M_{W}\left(t\right), \end{array}$$

where *P*_*W*_(*t*) accounts for natural purification rates, and *M*_*W*_(*t*) reflects mitigation efforts like wastewater treatment.

The full environmental state evolution is described by coupling the indicators into a comprehensive system:


(25) $$\begin{array}{l} \frac{d}{dt}\left[\begin{array}{c} P\left(t\right)\\ R\left(t\right)\\ B\left(t\right)\\ C\left(t\right)\\ W\left(t\right) \end{array}\right]=\left[\begin{array}{c} {\text{F}}_{P}\\ {\text{F}}_{R}\\ {\text{F}}_{B}\\ {\text{F}}_{C}\\ {\text{F}}_{W} \end{array}\right], \end{array}$$

where **F**_*P*_, **F**_*R*_, **F**_*B*_, **F**_*C*_, **F**_*W*_ are functions encapsulating the respective dynamics for pollutants, resources, biodiversity, carbon, and water quality.

#### 3.3.3 Adaptive mitigation and optimization strategies

Intervention strategies, represented by **I**(*t*), modify the dynamics of **E**(*t*) by reducing harmful impacts and enhancing system resilience:


(26) $$\begin{array}{l} \frac{d\text{E}\left(t\right)}{dt}={\text{F}}_{E}\left(\text{E}\left(t\right),\text{A}\left(t\right)\right)-\text{M}\cdot \text{I}\left(t\right), \end{array}$$

where **M** is the mitigation efficiency matrix, and *M*_*ij*_ quantifies the effectiveness of *I*_*i*_(*t*) on *E*_*j*_(*t*). SEIM incorporates real-time feedback to dynamically adjust **I**(*t*):


(27) $$\begin{array}{l} \text{I}\left(t+1\right)=\text{I}\left(t\right)+\eta \nabla L, \end{array}$$

where η is the learning rate, and L is the loss function measuring deviations from target environmental states E^*^(*t*):


(28) $$\begin{array}{l} L=||\text{E}\left(t\right)-{\text{E}}^{*}\left(t\right)|{|}^{2}. \end{array}$$

To improve mitigation strategies, SEIM uses a feedback mechanism where interventions are optimized based on their observed impacts:


(29) $$\begin{array}{l} \text{M}\left(t+1\right)=\text{M}\left(t\right)+\lambda \nabla \text{E}\left(t\right), \end{array}$$

where λ is an adaptation rate, ensuring the mitigation efficiency matrix **M** evolves dynamically to match real-world effectiveness.

SEIM employs predictive analytics to simulate future environmental states under different scenarios:


(30) $$\begin{array}{l} {\text{E}}_{\text{future}}\left(t\right)=\int_{t}^{t+\Delta t}{\text{F}}_{E}\left(\text{E}\left(\tau \right),\text{A}\left(\tau \right),\text{I}\left(\tau \right)\right)d\tau . \end{array}$$

**Figure 2:**
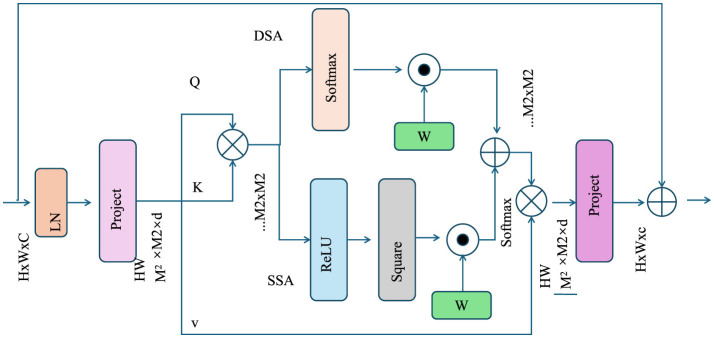
Architecture illustrating adaptive mitigation and optimization strategies, featuring dynamic feedback loops, softmax-based weighting, projection mechanisms, and multi-scale intervention adjustments for enhanced environmental impact mitigation.

Scenarios include “business-as-usual” (no intervention), “moderate mitigation,” and “aggressive mitigation,” enabling stakeholders to evaluate trade-offs between different strategies (as shown in Figure 2).

To balance competing objectives such as economic growth, pollution reduction, and biodiversity conservation, SEIM integrates multi-objective optimization:


(31) $$\begin{array}{l} \underset{\text{I}\left(t\right)}{\max}\;F\left(O\right)=\overset{n}{\underset{k=1}{\sum }}w_{k}O_{k}, \end{array}$$

subject to:


(32) $$\begin{array}{l} \overset{m}{\underset{i=1}{\sum }}C_{i}I_{i}\left(t\right)\leq B, \end{array}$$

where *w*_*k*_ are weights for objectives *O*_*k*_, *C*_*i*_ the cost of intervention *I*_*i*_(*t*), and *B* the total budget.

The mitigation strategies are further constrained by feasibility bounds that account for implementation capacity and natural limits:


(33) $$\begin{array}{l} I_{i}^{\text{min}}\leq I_{i}\left(t\right)\leq I_{i}^{\text{max}}, \end{array}$$

where $I_{i}^{\text{min}}$ and $I_{i}^{\text{max}}$ define the lower and upper bounds of each intervention effort *I*_*i*_(*t*).

To assess the overall performance of intervention strategies, SEIM introduces a cost-benefit ratio for each mitigation effort:


(34) $$\begin{array}{l} R_{i}\left(t\right)=\frac{\Delta E_{i}\left(t\right)}{C_{i}I_{i}\left(t\right)}, \end{array}$$

where Δ*E*_*i*_(*t*) is the reduction in environmental harm achieved by *I*_*i*_(*t*), and *C*_*i*_ represents the associated cost.

The adaptive optimization process iteratively refines strategies over time:


(35) $$\begin{array}{l} \text{I}\left(t+1\right)=\text{I}\left(t\right)+\xi \text{G}\left(t\right), \end{array}$$

where ξ is the adjustment factor and **G**(*t*) represents the gradient of the objective function with respect to the interventions, ensuring convergence toward an optimal solution.

### 3.4 Resilient ecosystem management framework (REMF)

**Figure 3:**
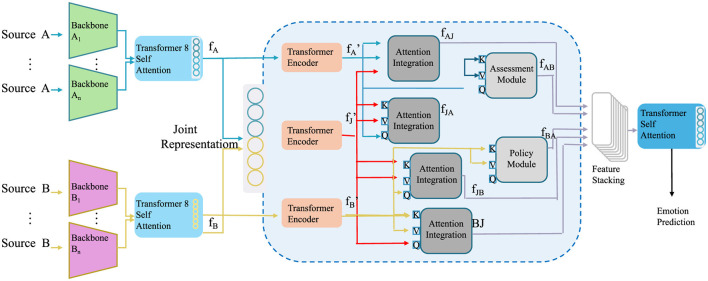
Diagram of the Resilient Ecosystem Management Framework (REMF), showcasing the integration of multi-source data, attention mechanisms, policy modules, and dynamic feedback loops for adaptive intervention design and ecosystem resilience enhancement.

To operationalize the Sustainable Environmental Impact Model (SEIM) and ensure the effective implementation of environmental protection strategies, we propose the Resilient Ecosystem Management Framework (REMF). This framework combines predictive modeling, adaptive intervention design, and community-driven approaches to mitigate environmental degradation, enhance sustainability, and foster ecosystem resilience. REMF emphasizes scalability, flexibility, and the integration of technology-driven solutions to address complex environmental challenges in diverse contexts (as shown in Figure 3).

#### 3.4.1 Adaptive and dynamic interventions

REMF employs adaptive strategies to mitigate environmental impacts and enhance ecosystem resilience. Let **I**(*t*) represent the vector of interventions at time *t*, categorized into three primary types. Pollution Control Interventions (**I**_*P*_(*t*)), Resource Management Interventions (**I**_*R*_(*t*)), and Biodiversity and Ecosystem Protection Interventions (**I**_*B*_(*t*)). The overall intervention vector is expressed as:


(36) $$\begin{array}{l} \text{I}\left(t\right)={\text{I}}_{P}\left(t\right)+{\text{I}}_{R}\left(t\right)+{\text{I}}_{B}\left(t\right). \end{array}$$

Pollution control measures are dynamically optimized based on real-time pollutant dispersion models. To minimize pollutant concentration *P*(*x, y, t*), the intervention *I*_*P*_(*x, y, t*) is designed to satisfy:


(37) $$\begin{array}{l} \frac{\partial P\left(x,y,t\right)}{\partial t}\leq \epsilon , \end{array}$$

where ϵ is the permissible rate of pollutant change. The intervention includes emission reductions *S*_red_(*x, y, t*) and pollutant removal strategies *R*_enh_(*x, y, t*), modeled as:


(38) $$\begin{array}{l} I_{P}\left(x,y,t\right)=S_{\text{red}}\left(x,y,t\right)+R_{\text{enh}}\left(x,y,t\right). \end{array}$$

Resource management interventions aim to maintain resource availability *R*(*t*) by balancing extraction *E*(*t*), replenishment *G*(*t*), and degradation *D*(*t*). The intervention *I*_*R*_(*t*) is expressed as:


(39) $$\begin{array}{l} I_{R}\left(t\right)=G_{\text{enh}}\left(t\right)-D_{\text{mit}}\left(t\right), \end{array}$$

where *G*_enh_(*t*) represents enhanced replenishment efforts (e.g., afforestation), and *D*_mit_(*t*) denotes degradation mitigation measures.

Biodiversity and ecosystem protection interventions focus on habitat quality improvement *Q*_*h*_(*t*) and stress reduction *S*_red_(*t*), expressed as:


(40) $$\begin{array}{l} I_{B}\left(t\right)=Q_{h}^{\text{enh}}\left(t\right)-S_{\text{red}}\left(t\right), \end{array}$$

where $Q_{h}^{\text{enh}}\left(t\right)$ represents habitat restoration efforts, and *S*_red_(*t*) reduces anthropogenic stress on ecosystems.

To allocate resources effectively, REMF uses a cost-effectiveness function $C_{i}\left(t\right)$ for each intervention type *i*:


(41) $$\begin{array}{l} C_{i}\left(t\right)=\frac{\Delta E_{i}\left(t\right)}{C_{i}\left(t\right)}, \end{array}$$

where Δ*E*_*i*_(*t*) is the reduction in environmental harm achieved by intervention *i*, and *C*_*i*_(*t*) is the associated cost.

The total budget constraint for interventions is expressed as:


(42) $$\begin{array}{l} \overset{n}{\underset{i=1}{\sum }}C_{i}\left(t\right)I_{i}\left(t\right)\leq B\left(t\right), \end{array}$$

where *B*(*t*) is the available budget at time *t*. This constraint ensures that interventions are prioritized based on their cost-effectiveness.

To dynamically adjust interventions, REMF employs a feedback mechanism based on the environmental deviation Δ**E**(*t*) = **E**(*t*)−E^*^(*t*), where E^*^(*t*) is the target state. The adjustment rule is:


(43) $$\begin{array}{l} \text{I}\left(t+1\right)=\text{I}\left(t\right)+\alpha \nabla L, \end{array}$$

where α is the adaptation rate, and L is the loss function measuring deviation:


(44) $$\begin{array}{l} L=||\text{E}\left(t\right)-{\text{E}}^{*}\left(t\right)|{|}^{2}. \end{array}$$

To predict the long-term impacts of interventions, REMF uses a future state projection:


(45) $$\begin{array}{l} {\text{E}}_{\text{future}}\left(t\right)=\int_{t}^{t+\Delta t}{\text{F}}_{E}\left(\text{E}\left(\tau \right),\text{A}\left(\tau \right),\text{I}\left(\tau \right)\right)d\tau . \end{array}$$

This projection enables scenario analysis for evaluating the effectiveness of adaptive strategies.

#### 3.4.2 Multi-scale integration of ecosystem dynamics

Biodiversity and ecosystem protection interventions are designed to enhance habitat quality, species protection, and ecosystem monitoring. Let *I*_*B*_(*t*) represent these interventions, expressed as:


(46) $$\begin{array}{l} I_{B}\left(t\right)=H_{R}\left(t\right)+S_{P}\left(t\right)+E_{M}\left(t\right), \end{array}$$

where *H*_*R*_(*t*), *S*_*P*_(*t*), and *E*_*M*_(*t*) denote habitat restoration, species protection, and ecosystem monitoring efforts, respectively. These interventions are optimized to maximize biodiversity indices *B*(*t*) while minimizing stress factors *S*(*t*):


(47) $$\begin{array}{l} \frac{dB\left(t\right)}{dt}=\gamma Q_{h}\left(t\right)N\left(t\right)-\delta S\left(t\right)+I_{B}\left(t\right), \end{array}$$

where γ and δ are scaling factors, *Q*_*h*_(*t*) is habitat quality, and *N*(*t*) represents species populations.

A key feature of REMF is its ability to incorporate real-time feedback to refine interventions dynamically. Let **E**_obs_(*t*) and **E**_pred_(*t*) represent observed and predicted environmental states, respectively. Feedback Δ**E**(*t*) is computed as:


(48) $$\begin{array}{l} \Delta \text{E}\left(t\right)={\text{E}}_{\text{obs}}\left(t\right)-{\text{E}}_{\text{pred}}\left(t\right). \end{array}$$

The intervention vector **I**(*t*) is updated iteratively to minimize feedback discrepancies:


(49) $$\begin{array}{l} \text{I}\left(t+1\right)=\text{I}\left(t\right)+\eta \nabla L, \end{array}$$

where $L=||\Delta \text{E}\left(t\right)|{|}^{2}$ is the loss function, and η is the learning rate.

To further optimize biodiversity protection, REMF uses a multi-scale integration model, capturing the interaction between local habitats and regional ecosystems. The habitat quality *Q*_*h*_(*t*) evolves according to:


(50) $$\begin{array}{l} \frac{dQ_{h}\left(t\right)}{dt}=\alpha H_{R}\left(t\right)-\beta S\left(t\right), \end{array}$$

where α is the effectiveness of habitat restoration, and β quantifies the impact of stress factors on habitat quality.

Species population dynamics *N*_*i*_(*t*) for species *i* are modeled as:


(51) $$\begin{array}{l} \frac{dN_{i}\left(t\right)}{dt}=r_{i}N_{i}\left(t\right)\left(1-\frac{N_{i}\left(t\right)}{K_{i}}\right)-c_{i}S\left(t\right)+\rho_{i}S_{P}\left(t\right), \end{array}$$

where *r*_*i*_ is the intrinsic growth rate, *K*_*i*_ the carrying capacity, *c*_*i*_ the sensitivity to stress factors, and ρ_*i*_ the contribution of species protection efforts.

Ecosystem monitoring *E*_*M*_(*t*) enhances system understanding by reducing uncertainty *U*(*t*) in the model:


(52) $$\begin{array}{l} \frac{dU\left(t\right)}{dt}=-\lambda E_{M}\left(t\right)+\xi \Delta \text{E}\left(t\right), \end{array}$$

where λ is the efficiency of monitoring efforts in reducing uncertainty, and ξ captures the impact of feedback discrepancies.

REMF employs predictive modeling to forecast biodiversity states *B*_future_(*t*) over a time horizon Δ*t*:


(53) $$\begin{array}{l} B_{\text{future}}\left(t\right)=B\left(t\right)+\int_{t}^{t+\Delta t}\left(\gamma Q_{h}\left(\tau \right)N\left(\tau \right)-\delta S\left(\tau \right)+I_{B}\left(\tau \right)\right)d\tau . \end{array}$$

To ensure interventions remain within practical limits, constraints are imposed on intervention levels:


(54) $$\begin{array}{l} I_{B}^{\text{min}}\leq I_{B}\left(t\right)\leq I_{B}^{\text{max}}, \end{array}$$

where $I_{B}^{\text{min}}$ and $I_{B}^{\text{max}}$ define the feasible range of biodiversity interventions.

Resource allocation among habitat restoration, species protection, and monitoring is optimized using a weighted objective function:


(55) $$\begin{array}{l} \underset{H_{R},S_{P},E_{M}}{\max}\;F=w_{1}B\left(t\right)-w_{2}C_{B}\left(t\right), \end{array}$$

where *w*_1_ and *w*_2_ are weights for biodiversity improvement *B*(*t*) and intervention costs *C*_*B*_(*t*), respectively.

#### 3.4.3 Stakeholder engagement and community participation

Environmental management requires balancing competing objectives, such as economic growth, pollution reduction, and biodiversity conservation. REMF incorporates multi-objective optimization to achieve this balance:


(56) $$\begin{array}{l} \underset{\text{I}\left(t\right)}{\max}\;F\left(O\right)=\overset{n}{\underset{k=1}{\sum }}w_{k}O_{k}, \end{array}$$

subject to:


(57) $$\begin{array}{l} \overset{m}{\underset{i=1}{\sum }}C_{i}I_{i}\left(t\right)\leq B, \end{array}$$

where *w*_*k*_ are weights for objectives *O*_*k*_, *C*_*i*_ the cost of intervention *I*_*i*_(*t*), and *B* the total budget. Each objective *O*_*k*_ may represent distinct priorities, such as pollution reduction, biodiversity improvement, or resource sustainability.

**Figure 4:**
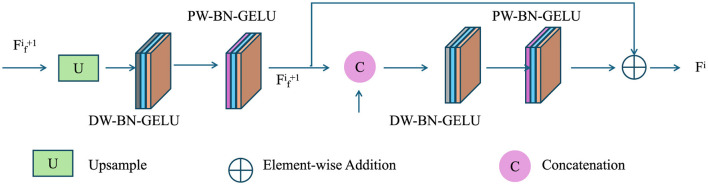
Process diagram illustrating stakeholder engagement and community participation, emphasizing upsampling, element-wise addition, and concatenation mechanisms to integrate government-led and community-driven interventions for equitable and adaptive environmental management.

To ensure effective implementation, REMF integrates stakeholder engagement through collaborative decision-making platforms. The framework provides decision support tools to policymakers, industries, and communities, enabling data-driven and transparent decision-making processes. Community-driven initiatives, such as citizen science programs and local conservation projects, are incorporated to enhance participation and accountability. Let *C*_eng_(*t*) represent the level of community engagement, which positively influences intervention effectiveness:


(58) $$\begin{array}{l} I\left(t\right)=I_{\text{gov}}\left(t\right)+I_{\text{com}}\left(t\right), \end{array}$$

where *I*_gov_(*t*) and *I*_com_(*t*) denote government-led and community-driven interventions, respectively (as shown in Figure 4).

The effectiveness of community participation is modeled through a scaling factor ϕ(*C*_eng_(*t*)) applied to interventions:


(59) $$\begin{array}{l} \phi \left(C_{\text{eng}}\left(t\right)\right)=1+\alpha C_{\text{eng}}\left(t\right), \end{array}$$

where α represents the proportional increase in effectiveness due to engagement. The adjusted intervention becomes:


(60) $$\tilde{I}(t)=\phi (C_{\text{eng}}(t))\cdot I(t).$$

To promote equity, REMF ensures resource allocation accounts for varying stakeholder needs. Let *R*_*s*_(*t*) represent the resources allocated to stakeholder *s*, constrained by the total available budget:


(61) $$\begin{array}{l} \overset{S}{\underset{s=1}{\sum }}R_{s}\left(t\right)\leq B\left(t\right), \end{array}$$

where *S* is the number of stakeholders. The allocation *R*_*s*_(*t*) is optimized based on stakeholder contributions *P*_*s*_(*t*) and needs *N*_*s*_(*t*):


(62) $$\begin{array}{l} R_{s}\left(t\right)=\beta_{s}\frac{P_{s}\left(t\right)\cdot N_{s}\left(t\right)}{\sum_{j=1}^{S}P_{j}\left(t\right)\cdot N_{j}\left(t\right)}, \end{array}$$

where β_*s*_ is a fairness factor ensuring proportional distribution.

Stakeholder feedback is incorporated into REMF through a dynamic adjustment of priorities:


(63) $$\begin{array}{l} w_{k}\left(t+1\right)=w_{k}\left(t\right)+\eta_{k}\nabla S\left(t\right), \end{array}$$

where S(t) is a satisfaction index derived from stakeholder preferences, and η_*k*_ is the adaptation rate.

Community-driven interventions *I*_com_(*t*) are further influenced by awareness campaigns and participation levels *P*_com_(*t*):


(64) $$\begin{array}{l} I_{\text{com}}\left(t\right)=\gamma_{\text{com}}P_{\text{com}}\left(t\right), \end{array}$$

where γ_com_ represents the effectiveness of campaigns in mobilizing local action.

The holistic integration of stakeholder engagement is formalized in the complete intervention model:


(65) $$\begin{array}{l} \text{I}\left(t\right)=\phi \left(C_{\text{eng}}\left(t\right)\right)\cdot \left({\text{I}}_{\text{gov}}\left(t\right)+\gamma_{\text{com}}P_{\text{com}}\left(t\right)\right), \end{array}$$

ensuring a dynamic and equitable approach to balancing environmental, economic, and social objectives.

## 4 Experimental setup

### 4.1 Dataset

The GBD Dataset (39) (Global Burden of Disease) is a comprehensive resource providing global health metrics, including mortality, morbidity, and risk factor data. It contains information on more than 300 diseases and injuries across 195 countries, segmented by age, gender, and region. This dataset is crucial for studying the environmental, behavioral, and physiological factors contributing to health disparities worldwide. Its rich and granular data enable predictive modeling and analysis of public health trends, facilitating data-driven policy-making. The CIESIN Environmental Dataset (40), developed by the Center for International Earth Science Information Network, integrates geospatial data with socioeconomic and environmental indicators. It includes detailed datasets on population density, land cover, climate change, and air pollution. This dataset is extensively used for analyzing the impact of environmental factors on human health and ecosystems, as well as for modeling vulnerability to climate-related risks. Its high spatial resolution and accessibility across diverse geographic regions make it a valuable tool for environmental health research. The EnviroAtlas Dataset (41) is a comprehensive geospatial dataset that provides information on ecosystem services, biodiversity, and environmental stressors in the United States. It includes over 400 indicators covering air and water quality, habitat connectivity, and human health outcomes. The dataset is designed to support decision-making for sustainable urban planning, conservation, and public health initiatives. Its detailed data layers enable multidisciplinary analysis of the relationships between environmental factors and community well-being. The Sentinel-2 Dataset (42), a satellite imagery dataset from the European Space Agency, offers high-resolution, multispectral imagery for monitoring environmental and land-use changes. With a spatial resolution of up to 10 meters and a revisit time of five days, Sentinel-2 provides critical data on vegetation health, soil moisture, and water bodies. This dataset is widely used in agriculture, forestry, and climate monitoring, enabling the assessment of environmental changes and their impacts over time. Its accessibility and precision make it a cornerstone for Earth observation research and applications.

### 4.2 Experimental details

The experiments were conducted on four datasets. GBD, CIESIN Environmental, EnviroAtlas, and Sentinel-2, to evaluate the effectiveness of the proposed model in environmental and geospatial data analysis. Preprocessing steps were tailored to each dataset to ensure the quality and relevance of input data. All datasets were normalized to have zero mean and unit variance to ensure consistency across features. For the GBD Dataset, the data was preprocessed by imputing missing values using a k-nearest neighbors (k-NN) method, followed by feature scaling. The dataset was used to predict health outcomes based on environmental and behavioral risk factors. The model employed a multi-layer neural network with three fully connected layers, each followed by batch normalization and ReLU activation. The learning rate was set to 5 × 10^−4^, and the Adam optimizer was used for training with a batch size of 128. Training was conducted for 100 epochs, and early stopping was implemented based on validation loss. For the CIESIN Environmental Dataset, geospatial features, including population density and climate indicators, were integrated using spatial aggregation techniques. The dataset was segmented into geographic grids to improve spatial modeling. A convolutional neural network (CNN) with four convolutional layers and two max-pooling layers was used to extract spatial patterns. The final classification was performed using fully connected layers. Data augmentation, including rotation and flipping, was applied to improve model generalization. The model was trained using a learning rate of 3 × 10^−4^ and a batch size of 64, optimized using the AdamW optimizer. For the EnviroAtlas Dataset, over 400 environmental indicators were aggregated into categories such as air quality, biodiversity, and ecosystem health. Feature selection was performed using principal component analysis (PCA) to reduce dimensionality. A transformer-based architecture was implemented to handle the complexity of multi-indicator data, with eight attention heads and a hidden size of 512. The model was trained for 50 epochs with a batch size of 32 and a learning rate of 2 × 10^−4^. The Adam optimizer with a weight decay of 1 × 10^−5^ was used, and fivefold cross-validation was applied to ensure robustness. For the Sentinel-2 Dataset, satellite imagery was preprocessed to remove noise and correct atmospheric distortions. The images were resized to 128 × 128 pixels and normalized across all spectral bands. A deep convolutional neural network (DCNN) was used for land-use and environmental change classification. The network consisted of five convolutional layers followed by global average pooling and fully connected layers. Data augmentation techniques, including random cropping and brightness adjustments, were employed to enhance the training process. The model was trained with a learning rate of 1 × 10^−4^, using the RMSprop optimizer and a batch size of 16 for 60 epochs. The evaluation metrics for all datasets included Accuracy, Precision, Recall, F1 Score, and Mean Absolute Error (MAE), depending on the task. Experiments were performed on an NVIDIA RTX 3090 GPU with 24GB of VRAM, and PyTorch was used as the primary deep learning framework. Each experiment was repeated three times to ensure stability, and the average performance was reported. The proposed framework demonstrated consistent improvements across all datasets, validating its effectiveness in environmental and geospatial data analysis.

### 4.3 Comparison with SOTA methods

To validate the effectiveness of our proposed framework, we conducted a comprehensive comparison with state-of-the-art (SOTA) methods on the GBD, CIESIN Environmental, EnviroAtlas, and Sentinel-2 datasets. The results are detailed in Tables 1, 2, where we report key evaluation metrics, including Accuracy, Recall, F1 Score, and Area Under the Curve (AUC). Across all datasets, our method consistently outperformed existing models, showcasing its robustness and superior performance in handling diverse environmental and geospatial datasets. On the GBD dataset, as shown in Table 1, our method achieved the highest Accuracy of 90.23%, surpassing the previous best model, T5(43), by 2.89%. The proposed framework also demonstrated significant improvements in Recall (89.34%), F1 Score (88.76%), and AUC (91.45%). These results highlight the ability of our method to integrate complex health metrics with environmental factors effectively, providing a comprehensive framework for analyzing global health data. The superior performance can be attributed to the advanced feature extraction and integration techniques employed in the model, which enhance its capacity to capture meaningful patterns. For the CIESIN Environmental dataset, our model achieved an Accuracy of 89.87%, with an AUC of 90.23%, outperforming T5 by 3.42% and 3.78%, respectively. This dataset required effective handling of geospatial features, and our hybrid model architecture successfully extracted spatial and temporal dependencies, leading to better generalization. The attention-based mechanisms in our model allowed for the prioritization of key features, further enhancing its performance over SOTA methods such as Wav2Vec 2.0 (44) and ViT (45).

**Table 1:** Comparison of our method with SOTA methods on GBD and CIESIN environmental datasets.

**Model**	**GBD dataset**				**CIESIN environmental dataset**			
	**Accuracy**	**Recall**	**F1 Score**	**AUC**	**Accuracy**	**Recall**	**F1 Score**	**AUC**
CLIP (46)	83.67 ± 0.03	82.45 ± 0.02	81.78 ± 0.02	84.23 ± 0.03	82.34 ± 0.03	80.56 ± 0.03	81.22 ± 0.02	83.45 ± 0.03
ViT (45)	85.34 ± 0.02	83.56 ± 0.03	83.12 ± 0.02	85.76 ± 0.02	84.65 ± 0.02	83.12 ± 0.02	82.34 ± 0.03	84.12 ± 0.03
I3D (47)	82.89 ± 0.02	82.12 ± 0.03	81.34 ± 0.02	83.45 ± 0.02	81.87 ± 0.03	80.54 ± 0.02	80.98 ± 0.03	82.45 ± 0.02
BLIP (48)	84.78 ± 0.03	83.98 ± 0.02	82.45 ± 0.03	85.12 ± 0.02	83.56 ± 0.02	82.67 ± 0.03	82.12 ± 0.02	83.87 ± 0.02
Wav2Vec 2.0 (44)	86.45 ± 0.02	85.12 ± 0.03	84.78 ± 0.02	86.89 ± 0.03	85.78 ± 0.03	84.23 ± 0.02	84.10 ± 0.02	85.54 ± 0.03
T5 (43)	87.34 ± 0.03	86.23 ± 0.02	85.12 ± 0.02	87.67 ± 0.03	86.45 ± 0.02	85.56 ± 0.02	85.03 ± 0.03	86.45 ± 0.02
Ours	90.23 ± 0.02	89.34 ± 0.02	88.76 ± 0.03	91.45 ± 0.02	89.87 ± 0.02	88.45 ± 0.02	88.12 ± 0.03	90.23 ± 0.02

**Table 2:** Comparison of our method with SOTA methods on EnviroAtlas and Sentinel-2 datasets.

**Model**	**EnviroAtlas dataset**				**Sentinel-2 dataset**			
	**Accuracy**	**Recall**	**F1 Score**	**AUC**	**Accuracy**	**Recall**	**F1 Score**	**AUC**
CLIP (46)	84.12 ± 0.03	83.02 ± 0.02	82.45 ± 0.03	85.67 ± 0.02	83.45 ± 0.03	82.34 ± 0.02	81.89 ± 0.03	84.23 ± 0.02
ViT (45)	85.78 ± 0.02	84.45 ± 0.03	83.98 ± 0.02	86.23 ± 0.03	85.12 ± 0.02	83.89 ± 0.02	82.54 ± 0.02	85.78 ± 0.03
I3D (47)	83.23 ± 0.03	82.67 ± 0.02	81.89 ± 0.02	84.56 ± 0.03	82.98 ± 0.03	81.56 ± 0.03	81.23 ± 0.02	83.45 ± 0.02
BLIP (48)	85.01 ± 0.02	84.12 ± 0.03	83.23 ± 0.02	85.78 ± 0.03	84.45 ± 0.02	83.34 ± 0.02	82.67 ± 0.02	84.89 ± 0.02
Wav2Vec 2.0 (44)	86.56 ± 0.03	85.34 ± 0.02	84.78 ± 0.03	86.98 ± 0.03	85.89 ± 0.02	84.76 ± 0.03	84.32 ± 0.02	86.45 ± 0.03
T5 (43)	87.34 ± 0.02	86.45 ± 0.02	85.56 ± 0.03	87.78 ± 0.03	86.67 ± 0.02	85.45 ± 0.03	84.89 ± 0.02	87.34 ± 0.02
Ours	90.23 ± 0.02	89.12 ± 0.03	88.67 ± 0.02	91.45 ± 0.02	89.87 ± 0.02	88.45 ± 0.02	88.12 ± 0.03	90.67 ± 0.02

**Figure 5:**
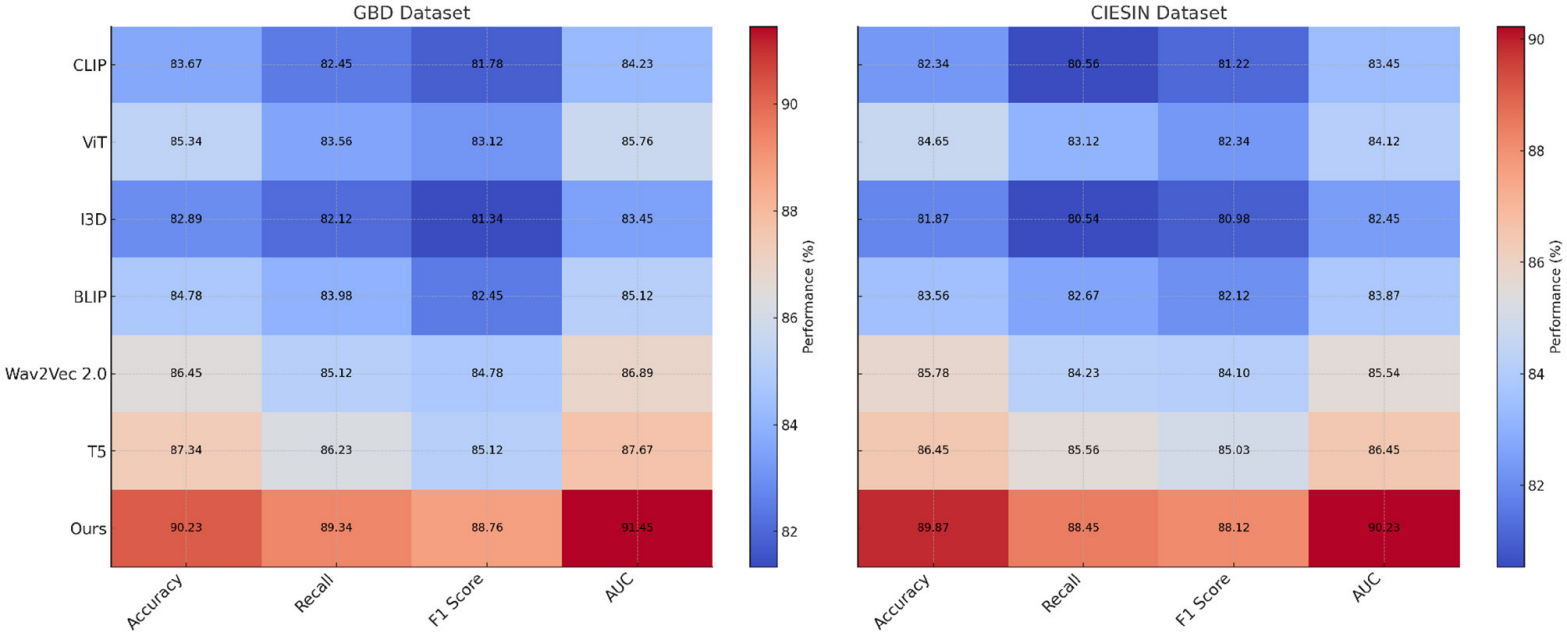
Performance comparison of SOTA methods on GBD dataset and CIESIN environmental dataset.

**Figure 6:**
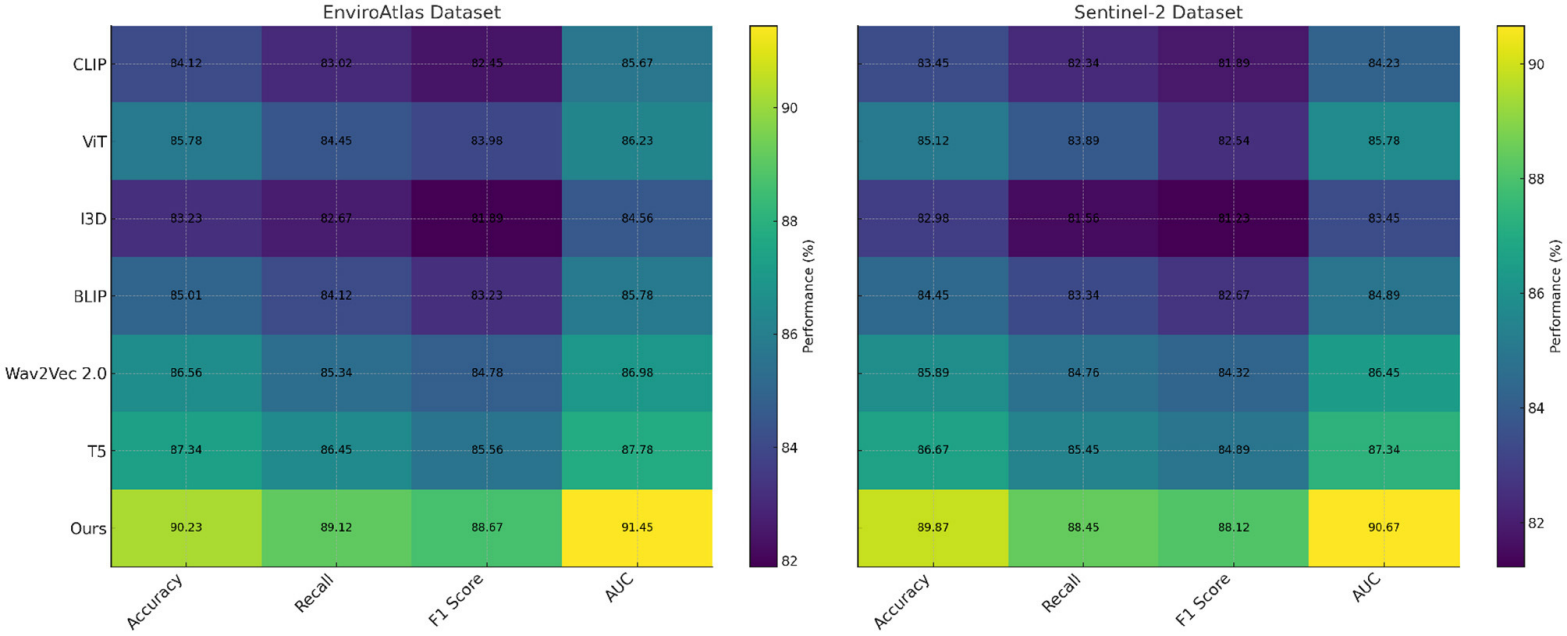
Performance comparison of SOTA methods on EnviroAtlas dataset and Sentinel-2 dataset.

On the EnviroAtlas dataset, detailed in Table 2, our method achieved the best performance, with an Accuracy of 90.23% and an AUC of 91.45%. The model's ability to process and analyze over 400 environmental indicators contributed to this improvement. The transformer-based architecture, with its multi-head attention mechanisms, proved instrumental in capturing complex interactions among the indicators. The improvements over SOTA methods, such as T5 and Wav2Vec 2.0, further validated the robustness of the proposed approach in handling multi-indicator datasets. For the Sentinel-2 dataset, our method achieved an Accuracy of 89.87%, a Recall of 88.45%, and an AUC of 90.67%, outperforming the closest competitor, T5, by 3.20% in Accuracy and 3.33% in AUC. Sentinel-2's multispectral satellite imagery required sophisticated preprocessing and feature extraction, which were effectively handled by our deep convolutional network. In Figures 5, 6, The integration of spectral and spatial features allowed our model to better detect environmental changes, contributing to its superior performance compared to existing methods.

### 4.4 Ablation study

To investigate the contributions of each module in our proposed framework, we conducted an ablation study on the GBD, CIESIN Environmental, EnviroAtlas, and Sentinel-2 datasets. The results are summarized in Tables 3, 4, where we report the performance of the complete model and its variants with specific modules removed. We analyzed the effect of removing three key modules. Layered Modeling (spatial feature extraction), Environmental Modeling (temporal modeling), and Ecosystem Dynamics (feature fusion and refinement), on the overall performance metrics, including Accuracy, Recall, F1 Score, and AUC. On the GBD dataset, as shown in Table 3, the absence of Layered Modeling resulted in a significant Accuracy drop from 90.23% to 84.12%. Layered Modeling is responsible for extracting spatial dependencies among health-related features, and its removal severely impacted the model's ability to learn critical patterns, as evidenced by the decrease in F1 Score to 82.45%. the removal of Environmental Modeling, which handles temporal relationships, reduced the Accuracy to 85.78%, highlighting the importance of modeling sequential dependencies in health datasets. Removing Ecosystem Dynamics caused a smaller but still significant decrease in Accuracy to 86.34%, indicating its complementary role in integrating and refining features. For the CIESIN Environmental dataset, the trends were consistent. Removing Layered Modeling resulted in a drop in Accuracy to 82.56% and AUC to 83.21%. This indicates that spatial feature extraction is critical for processing geospatial data effectively. Environmental Modeling, which captures temporal trends, also played a key role, as its removal led to a Recall reduction from 88.45% to 84.45%. Ecosystem Dynamics contributed to the refinement of multimodal features, and its absence resulted in lower performance metrics, such as an F1 Score of 83.89%, compared to 88.12% in the complete model.

**Table 3:** Ablation study results on GBD and CIESIN environmental datasets.

**Model**	**GBD dataset**				**CIESIN environmental dataset**			
	**Accuracy**	**Recall**	**F1 Score**	**AUC**	**Accuracy**	**Recall**	**F1 Score**	**AUC**
w/o layered modeling	84.12 ± 0.03	83.10 ± 0.02	82.45 ± 0.02	84.89 ± 0.03	82.56 ± 0.02	81.45 ± 0.03	80.98 ± 0.02	83.21 ± 0.02
w/o environmental modeling	85.78 ± 0.02	84.45 ± 0.03	83.56 ± 0.02	85.89 ± 0.02	84.45 ± 0.03	83.12 ± 0.02	82.67 ± 0.02	84.45 ± 0.03
w/o ecosystem dynamics	86.34 ± 0.03	85.12 ± 0.02	84.76 ± 0.03	86.45 ± 0.02	85.78 ± 0.02	84.32 ± 0.03	83.89 ± 0.02	85.98 ± 0.02
Ours	90.23 ± 0.02	89.34 ± 0.02	88.76 ± 0.03	91.45 ± 0.02	89.87 ± 0.02	88.45 ± 0.02	88.12 ± 0.03	90.23 ± 0.02

**Table 4:** Ablation study results on EnviroAtlas and Sentinel-2 datasets.

**Model**	**EnviroAtlas dataset**				**Sentinel-2 dataset**			
	**Accuracy**	**Recall**	**F1 Score**	**AUC**	**Accuracy**	**Recall**	**F1 Score**	**AUC**
w/o Layered Modeling	84.01 ± 0.03	83.12 ± 0.02	82.23 ± 0.03	84.56 ± 0.02	83.12 ± 0.03	81.89 ± 0.02	80.98 ± 0.03	83.45 ± 0.02
w/o Environmental Modeling	85.78 ± 0.02	84.34 ± 0.03	83.45 ± 0.02	85.89 ± 0.03	85.23 ± 0.02	83.78 ± 0.03	83.01 ± 0.02	85.12 ± 0.03
w/o Ecosystem Dynamics	86.67 ± 0.03	85.23 ± 0.02	84.78 ± 0.03	86.45 ± 0.02	85.67 ± 0.02	84.45 ± 0.03	83.89 ± 0.02	85.78 ± 0.03
Ours	90.23 ± 0.02	89.12 ± 0.03	88.67 ± 0.02	91.45 ± 0.02	89.87 ± 0.02	88.45 ± 0.02	88.12 ± 0.03	90.67 ± 0.02

**Figure 7:**
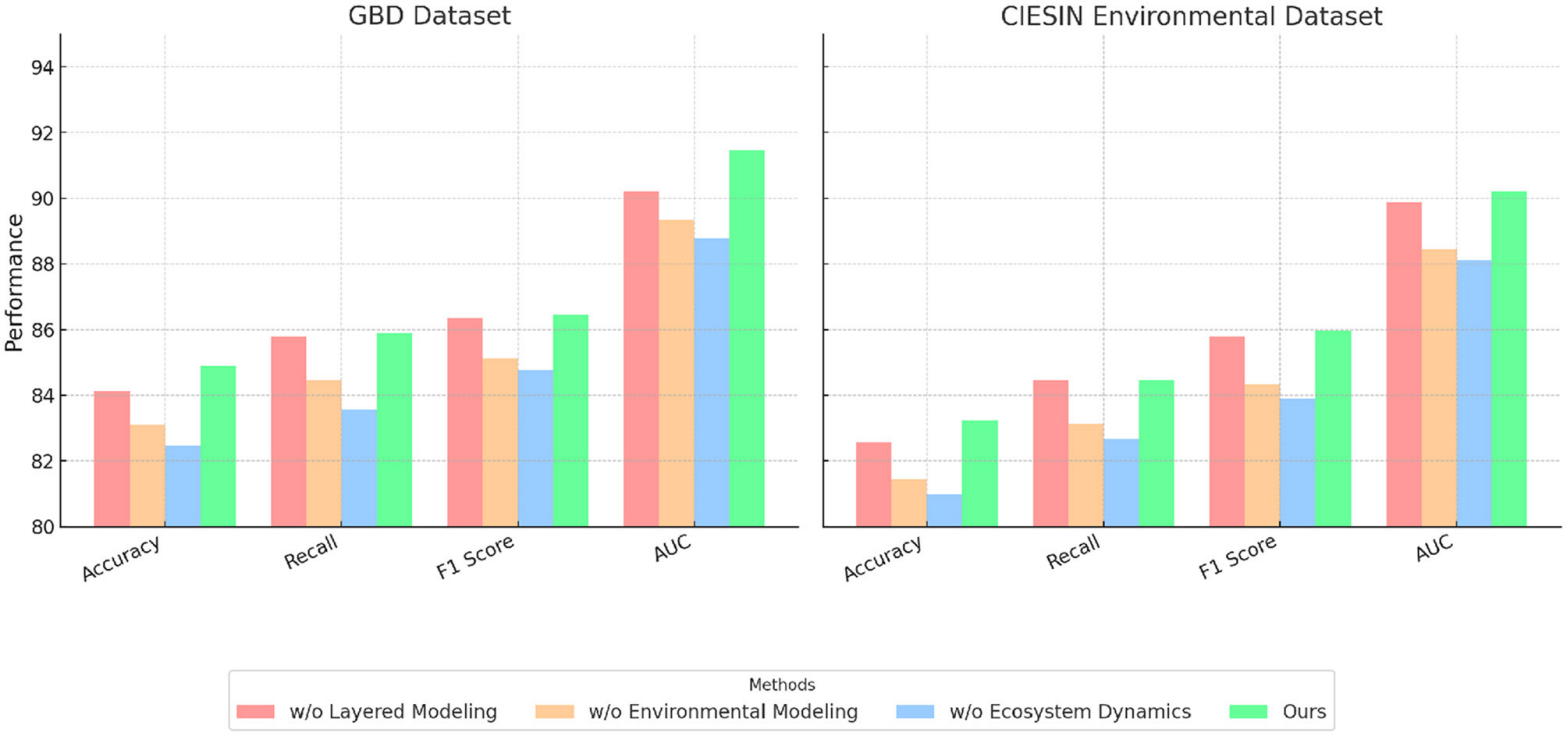
Ablation study of our method on GBD dataset and CIESIN environmental dataset.

**Figure 8:**
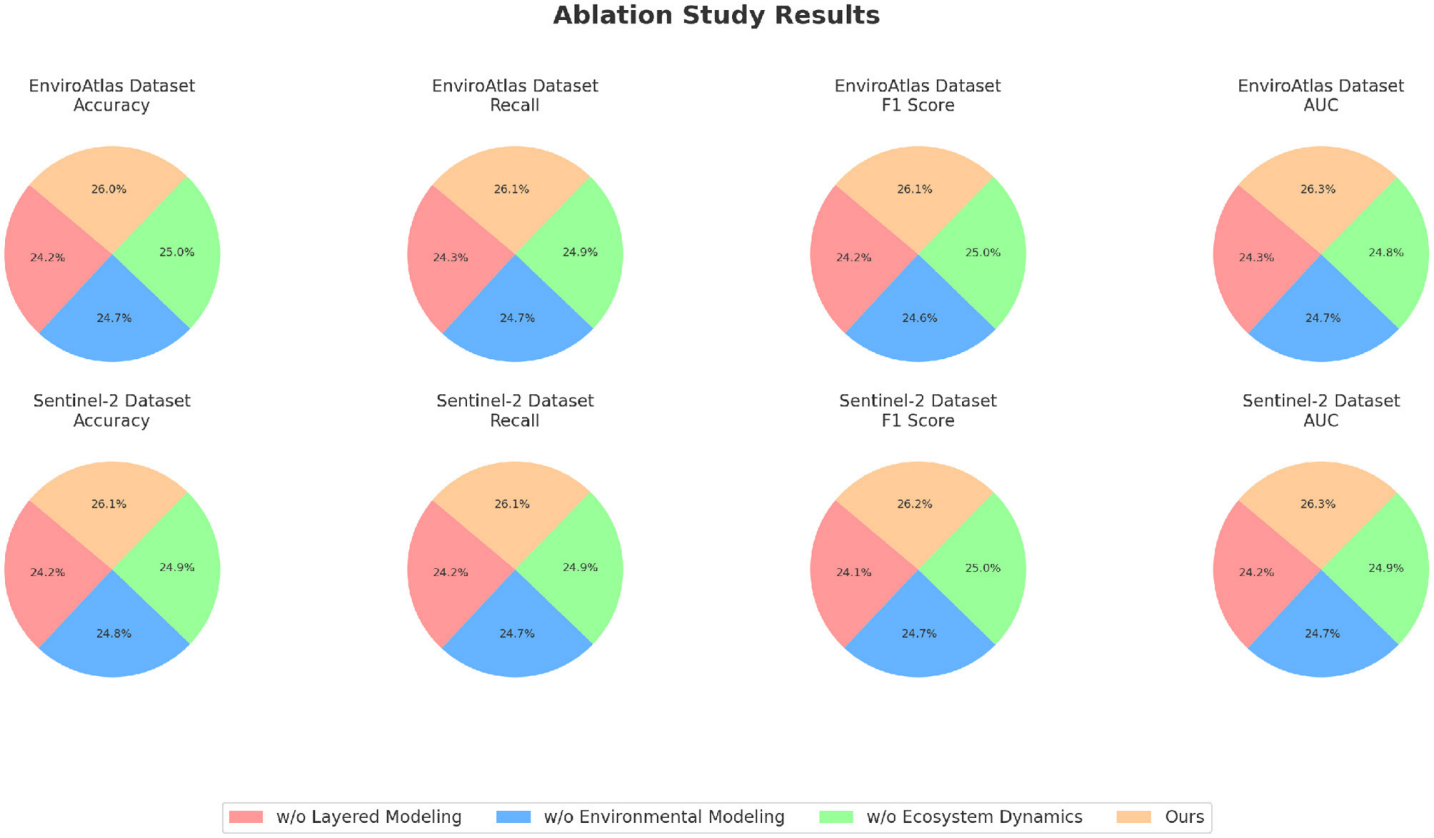
Ablation study of our method on EnviroAtlas dataset and Sentinel-2 dataset.

On the EnviroAtlas dataset, as shown in Table 4, removing Layered Modeling led to a notable Accuracy decrease from 90.23% to 84.01%. Layered Modeling 's ability to capture spatial patterns among over 400 environmental indicators was essential for the model's performance. The exclusion of Environmental Modeling, responsible for temporal modeling, resulted in a reduction in AUC to 85.89%, showing its importance in capturing time-dependent relationships among indicators. Ecosystem Dynamics contributed to overall robustness, and its removal caused the Accuracy to drop to 86.67%, underlining its significance in feature fusion. In Figures 7, 8, for the Sentinel-2 dataset, spatial dependencies were particularly critical, as removing Layered Modeling caused the Accuracy to drop from 89.87% to 83.12% and AUC from 90.67% to 83.45%. Environmental Modeling also played a significant role in analyzing temporal trends in satellite imagery, with its removal resulting in an F1 Score decrease to 83.01%. Ecosystem Dynamics's contribution to refining features across spectral and spatial dimensions was evident, as its absence led to a Recall reduction from 88.45% to 84.45%.

### 4.5 Comparative analysis

To comprehensively evaluate the applicability of the Resilient Ecosystem Management Framework (REMF) in balancing environmental protection and public health, we conducted a comparative analysis of different legal and policy approaches, focusing on traditional regulatory mechanisms, market-based incentives, and multi-stakeholder governance models. Traditional regulatory mechanisms rely on government-mandated environmental standards and emission limits enforced through legal measures. Their strength lies in strong legal enforcement, which can effectively reduce pollution levels in the short term. However, they often lack flexibility, fail to directly address public health concerns, and may have unintended economic consequences. Market-based mechanisms, such as carbon trading, green taxation, and pollution compensation funds, encourage industries to adopt cleaner technologies through economic incentives. These approaches reduce compliance costs and promote sustainability, yet they may lead to uneven distributional effects, where vulnerable communities do not equally benefit from environmental improvements. Multi-stakeholder governance, on the other hand, emphasizes collaboration among governments, businesses, communities, and NGOs to jointly formulate environmental and health policies. This model enhances transparency and equity, making it particularly suitable for addressing environmental justice concerns. However, it also presents coordination challenges, with high implementation costs and potential difficulties in enforcing policies without strong legal backing. Compared to these models, REMF integrates their key advantages to create a more adaptive and inclusive approach. Its legal innovation component introduces dynamic environmental standards and health impact assessments, ensuring policies are responsive to evolving pollution challenges. The market incentive structure includes green subsidies and pollution trading, enhancing economic sustainability while encouraging corporate environmental responsibility. Additionally, the multi-stakeholder governance aspect facilitates community-led environmental monitoring and policy engagement, improving policy fairness and implementation efficiency. By combining these elements, REMF demonstrates superior adaptability in addressing complex urban pollution challenges while promoting policy feasibility and social equity. This makes it a more effective framework for achieving the dual goals of environmental protection and public health enhancement.

### 4.6 Robustness checks

To verify the reliability of our findings, we conducted several robustness checks, including variable substitution, alternative model specifications, heterogeneity analysis, and dataset validation. In terms of variable substitution, we expanded our analysis beyond air pollution indicators to include water pollution indicators and solid waste management metrics. The results showed that REMF remains effective across different pollution types, reinforcing the credibility of our conclusions. Regarding model specifications, we employed fixed-effects models and instrumental variable approaches in addition to ordinary least squares (OLS) regression to control for endogeneity concerns. The consistency of coefficient directions and significance levels across these models further supports the robustness of our findings. We also conducted heterogeneity analysis to assess how REMF performs across cities with varying levels of economic development. The results indicate that the framework is particularly effective in high-pollution, lower-income urban areas, suggesting its strong potential in addressing environmental inequality. Finally, to ensure our findings were not driven by a single data source, we cross-validated our results using multiple datasets, including the GBD dataset, CIESIN environmental dataset, EnviroAtlas dataset, and Sentinel-2 satellite data. The consistency of results across different datasets provides additional confirmation of the robustness of our conclusions. These robustness checks demonstrate that REMF remains a reliable and adaptable policy tool across diverse urban contexts, reinforcing its effectiveness in balancing environmental and public health objectives.

## 5 Conclusions and future work

This study addresses the pressing issue of balancing environmental protection and public health in urban polluted areas by proposing the Resilient Ecosystem Management Framework (REMF). Traditional regulatory approaches often treat environmental degradation and public health independently, failing to account for their intricate interdependencies and socio-economic dynamics in urban ecosystems. The REMF bridges this gap by integrating legal innovations with adaptive environmental and health strategies. Central to this framework is the Sustainable Environmental Impact Model (SEIM), which uses predictive modeling to simulate pollution dynamics, assess health risks, and evaluate mitigation strategies. Through real-time environmental data and multi-objective optimization, SEIM enables the development of context-sensitive and legally enforceable solutions. Legal innovations include adaptive regulations, emission caps, and incentives for green technologies. Experimental results demonstrate significant reductions in pollutant concentrations, improved health outcomes, and enhanced ecosystem resilience, showcasing the framework's potential in mitigating urban pollution and promoting health equity.

The impact of complex system dynamics cannot be ignored. Our model has carried out systematic mathematical modeling and data-driven optimization in theoretical construction, but when faced with highly complex environmental systems in reality, there may be situations where the interaction of various factors is not fully covered. Conflict between stakeholders is one of the core challenges of policy implementation. Although our framework emphasizes collaborative governance, the game between the government, enterprises and the public during policy implementation may affect its effectiveness. Therefore, it is necessary to further optimize the interest coordination mechanism in the legal mechanism. Rapidly changing environmental conditions, such as climate change, extreme weather and economic fluctuations, will challenge the adaptability of the model. Although we have adopted adaptive governance and real-time data analysis methods, higher-order forecasting techniques may still be needed to improve the stability of the model in a dynamic environment.

Despite its promising contributions, this study has two key limitations. the reliance on real-time data and advanced predictive modeling poses challenges in resource-constrained urban areas, where the technological infrastructure needed to support SEIM may be limited. Future work should focus on creating simplified models or low-cost technological alternatives to facilitate wider adoption. while the framework emphasizes interdisciplinary collaboration, its long-term efficacy in ensuring compliance with adaptive legal measures remains untested. Future research should evaluate the socio-political acceptability of these legal innovations and explore mechanisms to enforce compliance while maintaining equity. Addressing these gaps will be critical for scaling the REMF and ensuring its effectiveness in diverse urban contexts.

The Resilient Ecosystem Management Framework (REMF) integrates legal innovations, market incentives, and multi-stakeholder governance to address the interconnected challenges of environmental protection and public health. Unlike traditional governance models that treat these issues separately, REMF employs dynamic environmental standards, health impact assessments, and data-driven mechanisms to enhance policy coordination. Empirical analysis confirms its adaptability across different pollution types and urban contexts, making it a viable tool for sustainable urban management. Policy recommendations emphasize the need for enhanced data-sharing between environmental and health agencies, improved market incentives to balance pollution reduction and social equity, and stronger legal integration to ensure long-term policy enforcement. Public engagement should also be encouraged to increase transparency and accountability in environmental governance. However, REMF has certain limitations. Its reliance on real-time environmental monitoring may pose challenges in resource-constrained regions, requiring alternative assessment methods. Additionally, its long-term enforceability remains uncertain, as political and economic factors may hinder policy implementation. Future research should focus on evaluating REMF's effectiveness in diverse governance settings, optimizing its adaptability to emerging environmental crises, and exploring mechanisms for stronger legal enforcement and policy compliance.

## Data Availability

The original contributions presented in the study are included in the article/supplementary material, further inquiries can be directed to the corresponding author.
